# Predictive Ability of Artificial Intelligence Algorithms in Pediatric Respiratory Disease Diagnosis Using Cough Sounds: A Systematic Review

**DOI:** 10.7759/cureus.88457

**Published:** 2025-07-21

**Authors:** Aya Abuelgasim Ibrahim Abdelhalim, Hanady ME M Osman, Sally Ibrahim Hafez Sadaka, Mohammed Abdelrahman Yousif Mohammed, Eman Eissa, Rasha Abdalla, Dilal Haroun Mohamad Hassan

**Affiliations:** 1 Emergency Medicine, Najran Armed Forces Hospital, Ministry of Defense Health Services, Najran, SAU; 2 Quality and Patient Safety, Najran Armed Forces Hospital, Ministry of Defense Health Services, Najran, SAU; 3 Pediatrics, Najran Armed Forces Hospital, Ministry of Defense Health Services, Najran, SAU; 4 Neonatal Intensive Care, Nottingham University Hospitals, Nottingham, GBR; 5 Pediatrics, Maidstone and Tunbridge Wells NHS Trust, Maidstone, GBR; 6 Pediatric Emergency, Dr. Sulaiman Al Habib Medical Group, Jeddah, SAU

**Keywords:** artificial intelligence, cough sound analysis, diagnostic accuracy, machine learning, pediatric respiratory diseases, systematic review

## Abstract

Respiratory diseases, including pneumonia, asthma, bronchiolitis, and croup, remain the leading causes of pediatric morbidity and mortality worldwide. Diagnostic challenges persist, especially in low-resource settings lacking specialized tools. Artificial intelligence (AI)-based analysis of cough sounds has emerged as a promising, noninvasive diagnostic alternative. This systematic review synthesizes evidence on the predictive ability of AI algorithms for diagnosing specific pediatric respiratory diseases using cough sounds, evaluating their diagnostic performance, clinical applicability, and methodological quality. Following the Preferred Reporting Items for Systematic Reviews and Meta-analyses (PRISMA) 2020 guidelines, six studies were included from 270 records identified in PubMed, Scopus, Web of Science, and IEEE Xplore databases. Eligible studies evaluated AI models such as logistic regression, convolutional neural networks (CNNs), support vector machines (SVMs), and hybrid feature-based approaches that combined acoustic and spectral features for disease classification. Techniques like wavelet-based feature extraction and late fusion, where outputs from multiple models are combined at the decision level, were reported to improve diagnostic accuracy. Sensitivity ranged from 82% to 94%, and specificity from 71% to 91% across studies, indicating high diagnostic potential, with some AI models outperforming conventional diagnostic methods such as the World Health Organization (WHO) clinical algorithms. Risk-of-bias assessment using Quality Assessment of Diagnostic Accuracy Studies-2 (QUADAS-2) revealed concerns in four studies (67%), mainly due to retrospective designs, small sample sizes (ranging from 65 to 585 participants), and lack of external validation. Study limitations included heterogeneous outcome definitions and insufficient reporting of model calibration. Overall, AI-driven cough sound analysis demonstrates significant promise as a rapid, scalable diagnostic tool for pediatric respiratory diseases, particularly in resource-limited settings. Future research should focus on prospective multicenter validation, transparent reporting of methodological details and performance metrics, and integration into clinical workflows to ensure safe and effective real-world implementation.

## Introduction and background

Respiratory diseases are leading causes of morbidity and mortality in children worldwide, imposing a heavy burden on healthcare systems, particularly in low- and middle-income countries [1]. Timely and accurate diagnosis is critical to ensure effective treatment and reduce complications [2]. However, conventional diagnostic methods, such as clinical examination, spirometry, chest radiography, and laboratory tests, are often unavailable in resource-constrained settings [3]. Additionally, young children frequently present with nonspecific symptoms and may struggle to cooperate with standard diagnostic procedures, further complicating accurate detection [4]. These challenges underscore the urgent need for noninvasive and accessible diagnostic tools that can support clinicians in such contexts.

Recent advancements in artificial intelligence (AI), particularly machine learning methods that enable computers to identify patterns from data, have generated interest in pediatric respiratory diagnosis [5]. For instance, studies by Abeyratne et al. and Porter et al. demonstrated that AI-based analysis of cough sounds can effectively detect conditions like pneumonia and asthma in children, suggesting its potential as a screening or triage tool [5,6]. AI models have also shown promising results in diagnosing adult respiratory diseases, such as chronic obstructive pulmonary disease and COVID-19, by processing complex acoustic and clinical data with high speed and accuracy [6].

Cough is one of the most common symptoms of respiratory illness and contains acoustic features that reflect changes in the airways and lungs [7]. AI algorithms, including traditional classifiers like support vector machines (SVMs) and advanced deep learning models such as convolutional neural networks (CNNs), can analyze these features to distinguish between different diseases [8]. Deep learning refers to AI techniques that use multiple computational layers to learn data representations automatically, often improving diagnostic accuracy compared to traditional models.

Despite these promising findings, the clinical applicability and generalizability of AI-based cough analysis remain uncertain [9]. Differences in study design, participant age groups, disease types assessed (e.g., pneumonia, asthma, bronchiolitis, croup), recording devices, and AI methodologies contribute to fragmented evidence requiring synthesis. Furthermore, while diagnostic accuracy metrics such as sensitivity and specificity are often reported, the predictive value of these models in real-world settings and their ethical implications, including data privacy and consent in pediatric populations, require further evaluation.

This systematic review aims to evaluate the diagnostic performance of AI algorithms in diagnosing pediatric respiratory diseases using cough sounds. By synthesizing evidence on AI model types, study characteristics, data acquisition methods, and reported diagnostic accuracy, this review seeks to highlight current advancements, identify methodological limitations, and inform future research for integrating AI-based cough analysis into pediatric care. Ultimately, it aspires to support the development of scalable and equitable diagnostic solutions for children worldwide.

## Review

Methodology

Study Design and Aim

This systematic review was conducted following the Preferred Reporting Items for Systematic Reviews and Meta-analyses (PRISMA) 2020 guidelines to ensure methodological rigor and transparency [10]. The aim was to evaluate the predictive ability of AI algorithms in diagnosing pediatric respiratory diseases using cough sounds.

Eligibility Criteria

Studies were included if they (1) focused on pediatric populations (0-18 years), (2) utilized AI or machine learning algorithms for respiratory disease diagnosis, (3) employed cough sounds as the primary input, and (4) reported diagnostic performance metrics such as sensitivity, specificity, or accuracy. Excluded were studies on adults, non-AI-based methods, reviews, conference abstracts, case reports, and editorials.

Information Sources and Search Strategy

A comprehensive search was conducted in PubMed, Scopus, Web of Science, and IEEE Xplore from database inception to May 2025. The strategy combined Medical Subject Headings (MeSH) and keywords related to AI, cough sounds, pediatric respiratory diseases, and diagnosis, using Boolean operators. Example terms included “artificial intelligence,” “machine learning,” “deep learning,” “cough sounds,” “pediatric,” “asthma,” and “pneumonia.” The search was limited to English-language articles. Additionally, reference lists of included studies were screened to identify further relevant publications. The full search strategy is provided in Table 4 in the appendices section. 

Study Selection

All retrieved records were imported into reference management software, and duplicates were removed. Two reviewers independently screened titles and abstracts, followed by full-text assessment against eligibility criteria. Disagreements were resolved through discussion or, if necessary, consultation with a third reviewer.

Data Extraction and Synthesis

Data were extracted using a structured form capturing study characteristics (e.g., country, sample size, setting), AI methodologies (model types, feature extraction techniques), diagnostic performance metrics (sensitivity, specificity, accuracy), and clinical applicability. “Standardized form” refers to this structured template designed for consistency across reviewers. Due to heterogeneity in study designs, AI algorithms, and reported outcomes, meta-analysis was not feasible. Therefore, a narrative synthesis was conducted, summarizing findings descriptively and identifying patterns in AI model performance, innovations, and study limitations.

Risk-of-Bias Assessment

The Quality Assessment of Diagnostic Accuracy Studies-2 (QUADAS-2) tool was used to assess risk of bias across four domains: patient selection, index test, reference standard, and flow and timing [11]. Two reviewers performed assessments independently, resolving disagreements by consensus. Cohen’s kappa statistic was calculated to assess inter-rater reliability, demonstrating substantial agreement. Results are presented in Table 3, indicating generally low risk, though some studies had unclear or high risk due to retrospective designs and small sample sizes.

Ethical Considerations

As this review analyzed previously published data, ethical approval was not required. Conflicts of interest were transparently reported (none declared), and all sources were cited appropriately.

Results

Studies Selection

**Figure 1:**
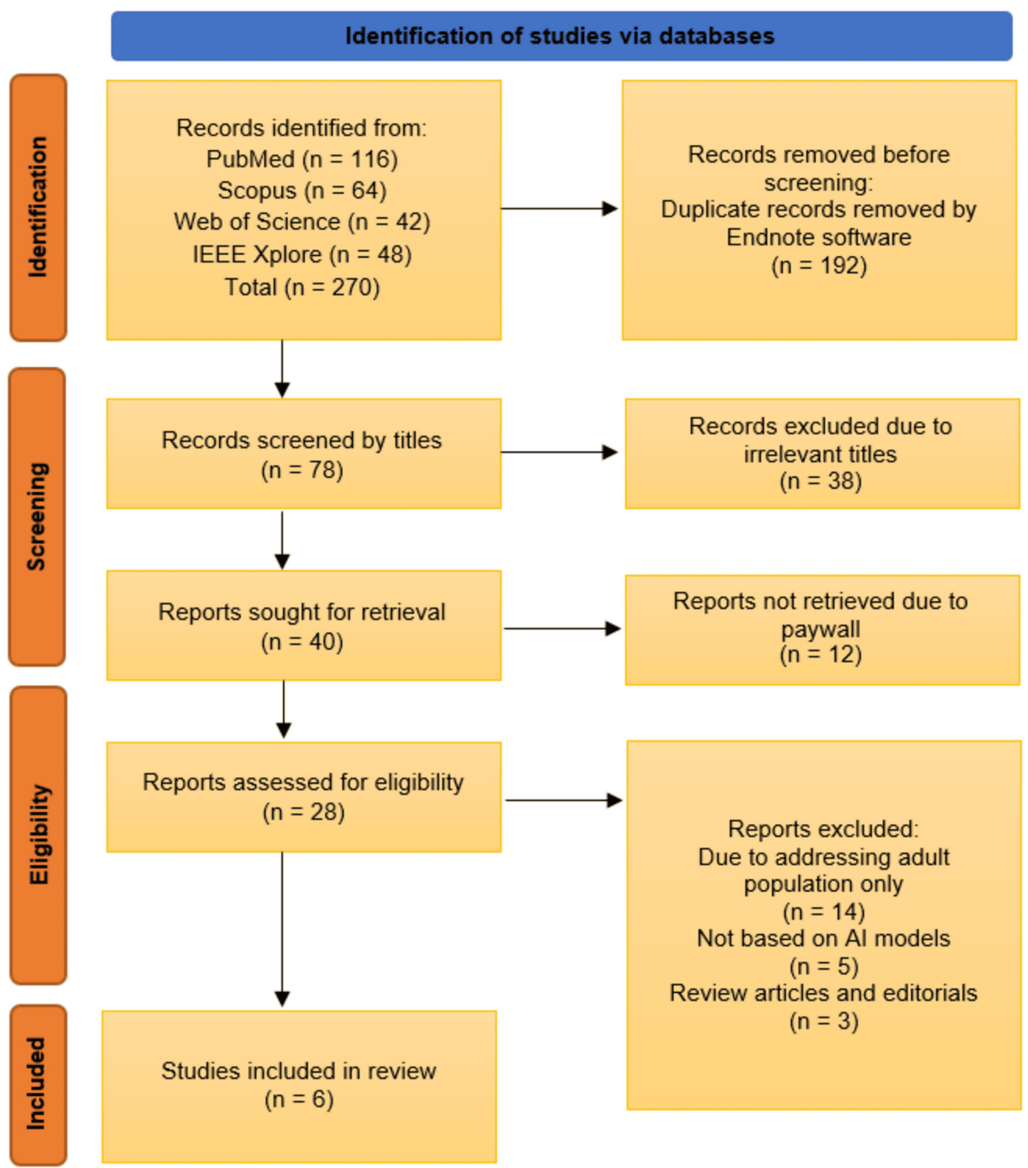
PRISMA flow diagram illustrating the study selection process PRISMA: Preferred Reporting Items for Systematic Reviews and Meta-analyses

The systematic review began with the identification of 270 records from four databases: PubMed (n = 116), Scopus (n = 64), Web of Science (n = 42), and IEEE Xplore (n = 48). After removing 192 duplicate records, 78 studies were screened by title, of which 38 were excluded for irrelevance. The remaining 40 full-text reports were sought for retrieval, but 12 were inaccessible due to paywall restrictions. Of the 28 reports assessed for eligibility, 14 were excluded for focusing solely on adult populations, five for not utilizing AI models, and three for being review articles or editorials. Ultimately, six studies met the inclusion criteria and were incorporated into the review (Figure 1) [12-17].

*Characteristics of Included Studies* 

The systematic review included six studies [12-17] that evaluated the diagnostic performance of AI algorithms in pediatric respiratory diseases using cough sounds. The studies were conducted across multiple countries, including Japan, China, Australia, and Indonesia, and employed diverse study designs, ranging from experimental diagnostic accuracy studies to observational studies with algorithm development and validation (Table 1). Sample sizes varied significantly, from 42 recordings in a study on pertussis [17] to 585 participants in a multicentre diagnostic accuracy study [16]. The respiratory diseases assessed included pneumonia, asthma, croup, bronchiolitis, bronchitis, and pertussis. 

**Table 1:** Key characteristics and main findings of the studies included in this systematic review

Author(s)	Country	Study design	Sample Size (n)	Respiratory disease(s) assessed	AI algorithm(s) used	Data source/recording device	Reference standard	Outcome measures	Key findings
Sharan et al. (2023) [12]	Japan and China	Experimental/diagnostic accuracy study	173	Pneumonia vs. other acute respiratory diseases	Multilayer perceptron (for denoising and classification); pretrained deep learning network for feature embedding	Cough sounds dataset; recording device not specified but implies smartphone use	Clinical diagnosis of pneumonia and other acute respiratory diseases	Sensitivity, specificity, signal-to-noise ratio improvement	Denoising improved SNR by 44%; cough segmentation sensitivity/specificity 91%/86%; pneumonia detection sensitivity/specificity 82%/71% using cough sounds alone; demonstrates feasibility as rapid diagnostic tool
Abeyratne et al. (2013) [13]	Australia and Indonesia	Observational study with algorithm development and validation	91	Pneumonia, bronchiolitis, asthma	Logistic regression classifier	Noncontact bedside microphones	Clinical diagnosis by a pediatric respiratory clinician	Sensitivity, specificity	Cough sound features plus fever presence achieved 94% sensitivity and 75% specificity for pneumonia diagnosis; performance better than WHO clinical algorithms; shows feasibility for remote areas
Kosasih et al. (2014) [14]	Indonesia	Diagnostic model development study	91 patients (815 cough sounds)	Pneumonia, asthma, bronchitis	Logistic regression classifier	Bedside microphone	Physician diagnosis aided by lab and radiological results	Sensitivity, Specificity	Sensitivity of 94% and specificity of 63% using wavelet features alone; improved to 94% sensitivity and 88% specificity when combined with other features; outperforms WHO criteria in resource-limited settings
Sharan et al. (2018) [15]	Australia	Experimental diagnostic development study	479 patients (364 training, 115 test)	Croup	Mathematical features inspired by the human auditory system: cochleagram, mel-frequency cepstral coefficients, feature combination, backward sequential feature selection	Cough sound recordings from patients with clinically diagnosed respiratory tract infections	Clinical diagnosis by physicians	Sensitivity, specificity	Automated cough sound analysis achieved 92.31% sensitivity and 85.29% specificity for distinguishing croup vs non-croup; shows significant improvement over previous methods
Porter et al. (2019) [16]	Australia	Diagnostic accuracy study	585	Asthma, pneumonia, lower respiratory tract disease, croup, bronchiolitis	Automated cough-sound analyser with up to five-symptom input	Cough sounds recorded in typical clinical environments; the first five coughs used	Consensus clinical diagnosis by a panel of paediatricians with hospital charts and investigations	Positive percent agreement (PPA), negative percent agreement (NPA)	High diagnostic agreement: asthma (PPA 97%, NPA 91%), pneumonia (PPA 87%, NPA 85%), lower respiratory tract disease (PPA 83%, NPA 82%), croup (PPA 85%, NPA 82%), bronchiolitis (PPA 84%, NPA 81%); supports use as high-level diagnostic aid
Sharan et al. (2021) [17]	Australia	Diagnostic model development and validation study	42 recordings (542 respiratory sound events)	Pertussis (whooping cough)	Convolutional neural networks (CNNs) with late fusion	Respiratory sound recordings; specific recording device not specified	Not explicitly described; likely clinical diagnosis of pertussis	Accuracy, AUC	Achieved 90.48% accuracy and AUC of 0.9501 for distinguishing pertussis vs non-pertussis; demonstrates feasibility of automated respiratory sound analysis for pertussis detection; potential for smartphone-based screening tool

The AI algorithms utilized in these studies were heterogeneous, encompassing logistic regression classifiers [13,14], multilayer perceptrons [12], CNNs [17], and custom mathematical feature-based approaches [15]. Data sources primarily consisted of cough sound recordings, often supplemented with clinical measurements such as fever [13]. Reference standards for diagnosis included clinical evaluations by physicians, radiological findings, and consensus panels, ensuring robust validation of the AI models. 

Diagnostic Performance of AI Algorithms

The diagnostic performance of AI algorithms in detecting pediatric respiratory diseases demonstrated high sensitivity and specificity across the included studies (Table 2). For pneumonia, Sharan et al. [12] reported a sensitivity of 91% and specificity of 86% for cough segmentation, while pneumonia detection achieved 82% sensitivity and 71% specificity. Similarly, Abeyratne et al. [13] achieved 94% sensitivity and 75% specificity by combining cough sound features with fever measurements, outperforming the World Health Organization (WHO) clinical algorithms in remote settings. Kosasih et al. [14] further reinforced these findings, with wavelet-based features yielding 94% sensitivity and 88% specificity, surpassing the WHO criteria. 

**Table 2:** Diagnostic performance of AI algorithms for pediatric respiratory disease using cough sounds LRTD: lower respiratory tract disease; AUC: area under the curve; MFCC: Mel-frequency cepstral coefficients; SNR: signal-to-noise ratio

Author(s)	Respiratory disease(s)	AI algorithm(s)	Performance metric	Sensitivity (%)	Specificity (%)	Accuracy (%)	AUC	Other reported metrics
Sharan et al. (2023) [12]	Pneumonia	Multi-layer perceptron (MLP) with cough denoising, segmentation, handcrafted features + deep learning embeddings	Cough segmentation: sensitivity 91%, specificity 86%; pneumonia detection: sensitivity 82%, specificity 71%	91 (segmentation), 82 (detection)	86 (segmentation), 71 (detection)	NR	NR	Denoising improved SNR by 44%
Abeyratne et al. (2013) [13]	Pneumonia (vs. other respiratory diseases like bronchiolitis, asthma)	Logistic regression classifier	Cough sound features (non-Gaussianity, Mel Cepstra); combined with simple measurements (e.g., fever)	94	75	NR	NR	Addition of fever further increased performance; performance superior to WHO clinical algorithms for remote regions
Kosasih et al. (2014) [14]	Pneumonia (vs. other respiratory diseases: asthma, bronchitis)	Logistic regression	Wavelet + other mathematical features	94%	88%	NR	NR	Outperforms WHO criteria
Sharan et al. (2018) [15]	Croup	Cochleagram + MFCC features with feature selection (not explicitly named but a custom classification approach)	Croup vs. non-croup classification	92.31%	85.29%	NR	NR	Experimental results show significant improvement over previous methods
Porter et al. (2019) [16]	Asthma (97/91%), pneumonia (87/85%), lower respiratory tract disease (83/82%), croup (85/82%), bronchiolitis (84/81%)	Automated cough-sound analyser with up to five-symptom input	Positive percent agreement/negative percent agreement	83-97	81-91	NR	NR	PPA and NPA per disease: asthma (97/91), pneumonia (87/85), LRTD (83/82), croup (85/82), bronchiolitis (84/81)
Sharan et al. (2021) [17]	Pertussis (whooping cough)	Convolutional neural networks (with mel-spectrogram, wavelet scalogram, cochleagram inputs, and late fusion)	Accuracy, AUC	NR	NR	90.48%	0.9501	Data augmentation, late fusion used to improve prediction; outperforms baseline techniques

For other respiratory conditions, AI algorithms also exhibited strong performance. Sharan et al. [15] achieved 92.31% sensitivity and 85.29% specificity in distinguishing croup from non-croup cases using cochleagram and mel-frequency cepstral coefficient (MFCC) features. Porter et al. [16] reported high positive and negative percent agreements for asthma (97%/91%), pneumonia (87%/85%), and other diseases, supporting the utility of automated cough sound analysis as a diagnostic aid. In pertussis detection, Sharan et al. [17] demonstrated an accuracy of 90.48% and an area under the curve (AUC) of 0.9501 using CNNs with late fusion, highlighting the potential for smartphone-based screening tools. 

Key Findings and Innovations

The studies collectively underscored the feasibility of AI-driven cough sound analysis as a rapid, noninvasive diagnostic tool for pediatric respiratory diseases. Innovations such as signal denoising [12], wavelet-based feature extraction [14], and late fusion techniques [17] significantly improved diagnostic accuracy. Notably, several studies emphasized the superiority of AI algorithms over traditional clinical methods, particularly in resource-limited settings [13,14]. 

Moreover, the integration of multimodal data, such as combining cough sounds with fever measurements [13], enhanced performance, suggesting that hybrid approaches may further optimize diagnostic outcomes. The studies also highlighted the potential for scalability, with smartphone-based recording devices proposed as practical tools for widespread implementation [16,17]. 

Risk-of-Bias Assessment

The risk-of-bias assessment, conducted using the QUADAS-2 tool, revealed that most studies demonstrated low to moderate risk of bias, though some limitations were identified. Prospective studies with clear methodologies, such as Sharan et al. [15] and Porter et al. [16], exhibited low risk across all domains, including patient selection, index test, reference standard, and flow/timing. However, Abeyratne et al. [13] and Kosasih et al. [14] were rated as having unclear risk due to potential retrospective recruitment and case-control design concerns, respectively. Sharan et al. [17] were assessed as high risk primarily due to their small sample size and insufficient detail on the reference standard. Despite these limitations, the index test (AI algorithm performance) was uniformly rated as low risk across all studies, as the analytical methods were well-described and independently validated. Overall, the findings support the reliability of the included studies, though caution is warranted when interpreting results from studies with a higher risk of bias (Table 3) [13,14,17].

**Table 3:** Risk of bias using QUADAS-2 tool QUADAS-2: Quality Assessment of Diagnostic Accuracy Studies-2

Study	Patient selection	Index test	Reference standard	Flow & timing	Overall risk of bias
Sharan et al. (2023) [12]	Low	Low	Low	Low	Low
Abeyratne et al. (2013) [13]	Unclear	Low	Low	Low	Unclear
Kosasih et al. (2014) [14]	Unclear	Low	Low	Low	Unclear
Sharan et al. (2018) [15]	Low	Low	Low	Low	Low
Porter et al. (2019) [16]	Low	Low	Low	Low	Low
Sharan et al. (2021) [17]	High	Low	Unclear	Low	High

Discussion

AI models demonstrated high diagnostic accuracy across studies, with sensitivity and specificity often exceeding those of traditional clinical methods, particularly in resource-limited settings. For example, Sharan et al. [12] reported 91% sensitivity and 86% specificity for pneumonia detection using cough segmentation, while Abeyratne et al. [13] achieved 94% sensitivity by combining cough features with fever measurements, outperforming WHO algorithms. These findings align with broader trends in AI diagnostics, where machine learning enhances disease detection when specialized infrastructure is lacking [18]. The ability to analyze cough sounds, a noninvasive and accessible biomarker, suggests potential for scalable, rapid pediatric respiratory disease diagnosis.

The reviewed studies employed diverse AI approaches tailored to specific diagnostic challenges. Logistic regression models, as used by Abeyratne et al. [13] and Kosasih et al. [14], offered interpretable results and performed robustly when combined with clinical data such as fever. More complex architectures, such as CNNs with late fusion, a method combining outputs from multiple models at the decision stage, demonstrated superior accuracy in distinguishing pertussis from other conditions, achieving an AUC of 0.9501 [17]. Studies also highlighted characteristic acoustic features: pertussis coughs with inspiratory whoops, pneumonia coughs with lower frequency wet sounds, and asthma coughs with short, dry bursts, reflecting different pathophysiological processes. This mirrors successes in other AI medical applications requiring complex pattern recognition [19]. Choosing the right model depends on context; simpler models may suffice when interpretability and efficiency are priorities, while deeper architectures may be used for higher diagnostic precision.

Integrating multimodal data emerged as a strategy to enhance diagnostic performance. Abeyratne et al. [13] improved pneumonia detection by combining cough sounds with fever data, aligning with literature advocating hybrid AI-clinical models [20]. Additionally, Kosasih et al. [14] utilized wavelet-based features, which involve analyzing signal frequency components at multiple scales, enhancing cough characterization, an approach effective in other audio-based diagnostics like snore analysis for sleep apnea [21].

Several studies emphasized the feasibility of smartphone-based AI platforms for real-world use. Sharan et al. [17] and Porter et al. [16] highlighted the potential for scalable implementation, crucial in settings with limited healthcare access. Beyond diagnosis, these tools may enable disease monitoring and early detection of exacerbations in chronic conditions, potentially reducing hospitalizations. This aligns with mobile health (mHealth) initiatives such as AI-powered stethoscopes for pulmonary assessments [22]. However, challenges remain, including standardizing recording protocols, minimizing environmental noise, and validating models across diverse populations, as most studies were conducted in specific regions (e.g., Australia, Indonesia), limiting generalizability.

Despite promising results, methodological variability was evident. While prospective studies such as Porter et al. [16] and Sharan et al. [15] showed low risk of bias, others faced limitations: Abeyratne et al. [13] and Kosasih et al. [14] had unclear recruitment methods, and Sharan et al. [17] involved small sample sizes. This reflects broader concerns in AI diagnostics research regarding retrospective designs and limited external validation [23]. Future studies should prioritize large, multicenter trials with standardized protocols to enhance reproducibility. Additionally, inconsistent reporting of performance metrics, some studies reported sensitivity and specificity, while others focused on accuracy or AUC, complicates comparisons. Adopting guidelines like the Standards for Reporting of Diagnostic Accuracy Study (STARD)-AI could address this [24].

The findings have broader implications for healthcare delivery. AI-based cough analysis could reduce diagnostic delays for diseases like pneumonia and pertussis, where timely treatment is critical, particularly in pediatric populations [25]. Studies used spectral features (frequency-based characteristics of cough sounds) and temporal features to differentiate wet coughs (linked to secretions) from dry coughs (airway irritation) and barky coughs (e.g., croup), enabling models to identify underlying causes accurately. The noninvasive nature of cough sound collection supports applications in remote monitoring and telemedicine, which gained relevance during the COVID-19 pandemic [26]. However, ethical considerations, such as data privacy, algorithmic bias, and the need for clinician oversight, remain essential to address before implementation.

This review has limitations. The small number of included studies (n = 6) restricts generalizability, and heterogeneity in study designs and AI approaches precluded meta-analysis. Most studies originated from high- and upper-middle-income countries (e.g., Australia, Japan), limiting insights into low-resource settings where these tools are most needed. Furthermore, long-term performance data are lacking, preventing assessment of sustained diagnostic accuracy over time. Future research should explore the integration of AI-based cough analysis into disease management pathways and public health surveillance. Finally, while QUADAS-2 assessed diagnostic bias, broader AI-specific methodological concerns, such as dataset imbalance and algorithm transparency, warrant attention.

## Conclusions

This analysis highlights the transformative potential of AI algorithms in diagnosing pediatric respiratory diseases using cough sounds. The included studies demonstrate that these tools can achieve high diagnostic accuracy, often outperforming conventional methods, and offer scalable solutions for resource-limited settings. However, the field must address key challenges, including methodological heterogeneity, limited generalizability, and the need for robust external validation, before widespread clinical adoption can be realized. Future research should focus on large-scale, diverse cohort studies and the development of standardized frameworks to ensure these technologies can fulfill their promise in improving global child health outcomes.

## Appendices

**Table 4:** Full search strategy for the databases used in this study

Database	Search strategy
PubMed	("artificial intelligence"[MeSH Terms] OR "machine learning"[MeSH Terms] OR "deep learning"[All Fields] OR "convolutional neural network"[All Fields] OR "support vector machine"[All Fields]) AND ("cough"[MeSH Terms] OR "cough sounds"[All Fields] OR "cough sound analysis"[All Fields]) AND ("pediatrics"[MeSH Terms] OR "child"[MeSH Terms] OR "children"[All Fields] OR "pediatric"[All Fields]) AND ("respiratory tract diseases"[MeSH Terms] OR "respiratory disease"[All Fields] OR "asthma"[MeSH Terms] OR "pneumonia"[MeSH Terms] OR "bronchiolitis"[All Fields] OR "croup"[All Fields]) AND ("diagnosis"[MeSH Terms] OR "diagnostic accuracy"[All Fields] OR "sensitivity and specificity"[MeSH Terms])
Scopus	TITLE-ABS-KEY ("artificial intelligence" OR "machine learning" OR "deep learning" OR "convolutional neural network" OR "support vector machine") AND TITLE-ABS-KEY ("cough" OR "cough sounds" OR "cough sound analysis") AND TITLE-ABS-KEY ("pediatric" OR "child" OR "children") AND TITLE-ABS-KEY ("respiratory disease" OR "asthma" OR "pneumonia" OR "bronchiolitis" OR "croup") AND TITLE-ABS-KEY ("diagnosis" OR "diagnostic accuracy" OR "sensitivity" OR "specificity")
Web of Science	TS=("artificial intelligence" OR "machine learning" OR "deep learning" OR "convolutional neural network" OR "support vector machine") AND TS=("cough" OR "cough sounds" OR "cough sound analysis") AND TS=("pediatric" OR "child" OR "children") AND TS=("respiratory disease" OR "asthma" OR "pneumonia" OR "bronchiolitis" OR "croup") AND TS=("diagnosis" OR "diagnostic accuracy" OR "sensitivity" OR "specificity")
IEEE Xplore	("All Metadata":"artificial intelligence" OR "All Metadata":"machine learning" OR "All Metadata":"deep learning" OR "All Metadata":"convolutional neural network" OR "All Metadata":"support vector machine") AND ("All Metadata":"cough" OR "All Metadata":"cough sounds" OR "All Metadata":"cough sound analysis") AND ("All Metadata":"pediatric" OR "All Metadata":"child" OR "All Metadata":"children") AND ("All Metadata":"respiratory disease" OR "All Metadata":"asthma" OR "All Metadata":"pneumonia" OR "All Metadata":"bronchiolitis" OR "All Metadata":"croup") AND ("All Metadata":"diagnosis" OR "All Metadata":"diagnostic accuracy" OR "All Metadata":"sensitivity" OR "All Metadata":"specificity")
